# Fore-Aft Asymmetry Improves the Stability of Trotting in the Transverse Plane: A Modeling Study

**DOI:** 10.3389/fbioe.2022.807777

**Published:** 2022-06-03

**Authors:** Mau Adachi, Shinya Aoi, Tomoya Kamimura, Kazuo Tsuchiya, Fumitoshi Matsuno

**Affiliations:** ^1^ Department of Mechanical Engineering and Science, Graduate School of Engineering, Kyoto University, Kyoto, Japan; ^2^ Department of Aeronautics and Astronautics, Graduate School of Engineering, Kyoto University, Kyoto, Japan; ^3^ Department of Electrical and Mechanical Engineering, Nagoya Institute of Technology, Nagoya, Japan

**Keywords:** fore-aft asymmetry, quadrupedal trotting, transverse dynamics, gait stability, simple model

## Abstract

Quadrupedal mammals have fore-aft asymmetry in their body structure, which affects their walking and running dynamics. However, the effects of asymmetry, particularly in the transverse plane, remain largely unclear. In this study, we examined the effects of fore-aft asymmetry on quadrupedal trotting in the transverse plane from a dynamic viewpoint using a simple model, which consists of two rigid bodies connected by a torsional joint with a torsional spring and four spring legs. Specifically, we introduced fore-aft asymmetry into the model by changing the physical parameters between the fore and hind parts of the model based on dogs, which have a short neck, and horses, which have a long neck. We numerically searched the periodic solutions for trotting and investigated the obtained solutions and their stability. We found that three types of periodic solutions with different foot patterns appeared that depended on the asymmetry. Additionally, the asymmetry improved gait stability. Our findings improve our understanding of gait dynamics in quadrupeds with fore-aft asymmetry.

## 1 Introduction

Quadrupedal mammals have fore-aft asymmetry in their body structure. For example, their fore and hind legs have not only different skeletal structures but also different masses and properties of muscles (Payne et al., 2005a, b; Williams et al., 2008a, b). During their locomotion, while the fore legs generate more braking forces than the hind legs, the hind legs do more propulsive forces than the fore legs (Lee et al., 1999; Bertram and Gutmann 2008). Furthermore, the fore and hind legs have different connections to the body; while the fore legs are suspended by muscles through the scapula, the hind legs are connected to the pelvis *via* skeletal articulation (Hildebrand and Goslow, 2001). In addition, the front part of the bodies of horses and dogs is heavier than the hind part because the front part has a head and neck and the thorax has higher density and larger mass than the abdomen (Buchner et al., 1997; Jones et al., 2018). To compensate for the asymmetric mass distribution, the forelegs generally support more of the body weight than the hind legs (Rollinson and Martin, 1981; Merkens et al., 1993; Lee et al., 1999). Additionally, horses, which have a long neck to increase fore-aft asymmetry, use not only their forelegs but also their thoracic muscles to support their weight (Payne et al.,. 2005b). These asymmetric body structures affect their walking and running dynamics. However, the effects of fore-aft asymmetry on quadrupedal locomotion remain largely unclear. To date, these effects have been investigated in the sagittal plane using both biological approaches (Lee et al., 2004; Lee, 2010) and modeling approaches (Zou and Schmiedeler, 2006; Yamada et al., 2022). For example, in a modeling study using a simple model, researchers demonstrated that the forward offset of the center of mass (COM) position reduces the stability of bounding gait in the sagittal plane (Zou and Schmiedeler, 2006). The effects of asymmetry are crucial not only in the sagittal plane but also in the transverse plane. However, few studies have investigated the effects in the transverse plane; thus, the effects remain unclear.

In this study, we investigate the effects of fore-aft asymmetry on trotting in the transverse plane using a simple model. Although quadrupedal mammals use various gaits, such as walking, trotting, and galloping, depending on their locomotion speed, trotting is widespread among quadrupedal mammals (Muybridge, 1957; Alexander and Jayes, 1983). In trotting, their four legs are used in two pairs, that is, the diagonal fore and hind legs, and these two pairs of legs touch the ground alternately (Hildebrand, 1965, 1968). During such trotting, quadrupedal mammals basically keep their bodies parallel to the ground unlike other gaits (Muybridge, 1957; Heglund et al., 1974; Dunbar et al., 2008). Although the diagonal touchdown generates moments to rotate the fore and hind parts of the body in opposite directions not only in the sagittal plane but also in the transverse plane, which makes it difficult to maintain their posture during trotting, quadrupedal mammals stabilize their body using trunk muscles (Schilling and Carrier, 2009). In our previous work (Adachi et al., 2020), we used a simple fore-aft symmetrical model in the transverse plane, which had two segmented bodies connected by a torsional joint with a torsional spring and four spring legs, and found that the appropriate stiffness in the body and legs produced stable trotting. However, the fore-aft asymmetry makes differences between the moments by the diagonal touchdown, which changes the gait characteristics and stability. To investigate the effects of fore-aft asymmetry on transverse dynamics in trotting, we extend our previous model to incorporate fore-aft asymmetry and examine the asymmetry effects on trotting from a dynamic viewpoint.

## 2 Materials and Methods

### 2.1 Model

**FIGURE 1:**
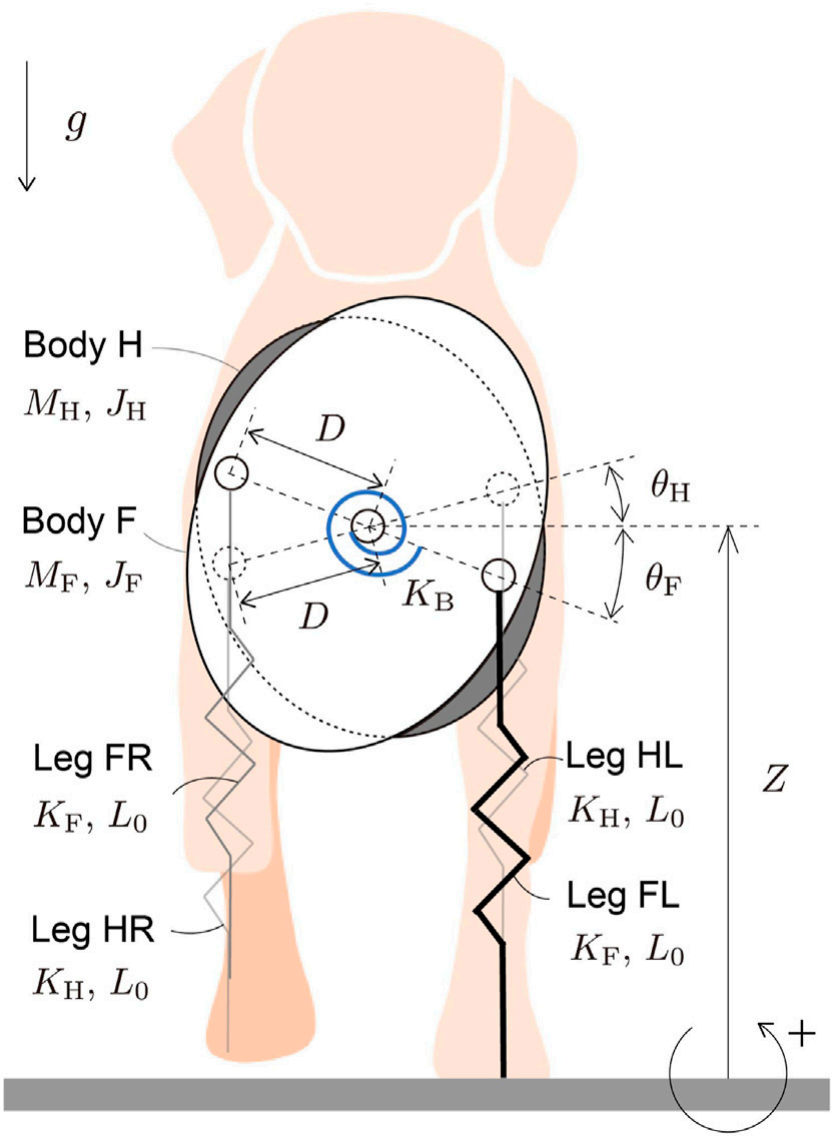
Our model composed of two rigid bodies and four massless springs. The bodies are connected at their COM by a joint with a torsional spring.

Each leg of a quadrupedal mammal has only 10% or less of the total mass (Buchner et al., 1997; Amit et al., 2009; Kilbourne and Hoffman, 2013). The main function of the legs is to produce reaction forces from the ground to support the body and can be represented by a spring (e.g., Full and Koditschek, 1999). Because stabilization of the body posture is crucial to generate stable gait, we focused on the dynamics of the body posture in the transverse plane and used massless springs for the legs in our model. Specifically, the model consists of two rigid bodies and four massless springs (Figure 1). The two rigid bodies represent the fore and hind parts of the body (Bodies F and H, respectively), and are connected by a joint at their COM. The four massless springs represent the legs (Legs FL, FR, HL, and HR). Legs *i*L and *i*R (*i* = F, H) are connected to Body *i* on the left and right sides, respectively. Because mediolateral ground reaction forces (GRFs) are much smaller than vertical forces during trotting of quadrupedal mammals (Merkens et al., 1993; Gillette and Angle, 2008), we ignore the horizontal dynamics of our model, as in previous studies (Berkemeier, 1998; De and Koditschek, 2018) and focus on the vertical and rotational movements of the bodies. *Z* is the vertical position of the COM of the bodies. *θ*_*i*_ (*i* = F, H) is the angle of Body *i* relative to the horizontal line. *L*_*ij*_ (*i* = F, H, *j* = L, R) is the length of Leg *ij*. The mass and moment of inertia around the COM of Body *i* (*i* = F, H) are *M*_*i*_ and *I*_*i*_, respectively. The body joint has a torsional spring with a spring constant of *K*_B_ that produces the body torsional movement. The body spring is at the equilibrium position when the bodies have the same posture (*θ*_F_ = *θ*_H_). The spring constants of the forelegs (Legs FL and FR) and hind legs (Legs HL and HR) are *K*_F_ and *K*_H_, respectively. All the legs have the same nominal length *L*_0_. The distance between the COM of the bodies and the root of the leg spring is *D* for both the fore and hind bodies. The gravitational acceleration is g .

When Leg *ij* (*i* = F, H, *j* = L, R) is in the air, it remains vertical and maintains the nominal length (*L*_*ij*_ = *L*_0_). When the tip touches the ground, the leg spring starts to compress to receive a GRF. When its length returns to the nominal length (*L*_*ij*_ = *L*_0_) after compression, the tip leaves the ground. Because touchdowns and liftoffs occur at the nominal length, our model is energy conservative.

The equations of motion of the model are given by

$$\left(M_{\text{F}}+M_{\text{H}}\right)\ddot{Z}+\;\underset{i\in I,\;j\in J}{\sum }\;F_{ij}+\left(M_{\text{F}}+M_{\text{H}}\right)g=0$$ (1a)

$$I_{i}{\ddot{\theta }}_{i}+\underset{j\in J}{\sum }F_{ij}D_{j}\cos\theta_{i}+K_{\text{B}}\left(\theta_{i}-\varphi_{i}\right)=0\;i=F,H,$$ (1b)

$I=\left\{F,H\right\},\;J=\left\{L,R\right\},\;\varphi_{\text{F}}=\theta_{\text{H}},\;\varphi_{\text{H}}=\theta_{\text{F}},\;D_{\text{L}}=D,\;D_{\text{R}}=-D$ , and

$$F_{ij}=\left\{\begin{array}{cc} K_{i}\left(L_{ij}-L_{0}\right)\; & stance\;phase\\ 0\; & swing\;phase \end{array}\right.\;i=F,H,\;j=L,R,$$

where *L*_*ij*_ = *Z* + *D*_*j*_ sin *θ*_*i*_. Leg *ij* touches the ground when its tip reaches the ground and leaves the ground when its length returns to the nominal length. These conditions are both given by

$$R_{ij}\left(Q\right)=Z+D_{j}\sin\theta_{i}-L_{0}=0\;i=F,H,\;j=L,R,$$ (2)

where $Q={\left[Z\;\theta_{\text{F}}\;\theta_{\text{H}}\;\dot{Z}\;{\dot{\theta }}_{\text{F}}\;{\dot{\theta }}_{\text{H}}\right]}^{\text{T}}$ .

To generalize the dynamics of the model, we non-dimensionalize the governing equations using the mass scale *M*_F_ + *M*_H_, length scale *D*, and time scale $\sqrt{D/g}$ . The dimensionless equations of motion are given by

$$\ddot{z}+\;\underset{i\in I,\;j\in J}{\sum }\;f_{ij}+1=0$$ (3a)

$$\mu_{i}{\ddot{\theta }}_{i}+\underset{j\in J}{\sum }d_{j}f_{ij}\cos\theta_{i}+k_{\text{B}}\left(\theta_{i}-\varphi_{i}\right)=0\;i=F,H,$$ (3b)

$z=\left(Z-L_{0}\right)/D,\;\tau =t/\sqrt{D/g}$ ,

$$f_{ij}=\left\{\begin{array}{cc} k_{i}\left(z+d_{j}\sin\theta_{i}\right)\; & stance\;phase\\ 0\; & swing\;phase \end{array}\right.\;i=F,H,\;j=L,R,$$

*μ*_*i*_ = *I*_*i*_/((*M*_F_ + *M*_H_)*D* ^2^), *k*_*i*_ = *K*_*i*_ *D*/((*M*_F_ + *M*_H_) g ) (*i* = F, H), *k*_B_ = *K*_B_/((*M*_F_ + *M*_H_) g *D*), *d*_L_ = 1, *d*_R_ = −1, and hereafter, $\dot{*}$ indicates the derivative of variable * with respect to *τ*. The dimensionless condition for the touchdown and liftoff of Leg *ij* is given by

$$r_{ij}\left(q\right)=z+d_{j}\sin\theta_{i}=0\;i=F,H,\;j=L,R,$$ (4)

where $q={\left[z\;\theta_{\text{F}}\;\theta_{\text{H}}\;\dot{z}\;{\dot{\theta }}_{\text{F}}\;{\dot{\theta }}_{\text{H}}\right]}^{\text{T}}$ .

### 2.2 Gait Assumptions

During trotting, the four legs work in two pairs. Specifically, the diagonal legs (Legs FL and HR, and Legs FR and HL) are paired. These two pairs touch the ground alternately. In this study, we focus on the motions during which one pair of legs touches and leaves the ground and then the other pair does the same. We assume that each leg touches the ground only once in a single gait cycle. Additionally, when one leg of a pair touches the ground, it never leaves the ground until the other leg of that pair touches the ground, that is, a double stance phase exists for each pair. We define the following four phases: flight (F), fore stance (FS), hind stance (HS), and double stance (DS) phases. In the flight phase, all the legs are in the air. In the fore (hind) stance phase, only the fore (hind) leg of a pair is in contact with the ground. In the double stance phase, both legs of a pair are in contact with the ground.

Because the model is left-right symmetric, the motion during which one pair touches and leaves the ground, and the motion during which the other pair touches and leaves the ground can be expressed using the same expression when the left and right sides of the model are reversed. Specifically, we use *q* ^+^ = *B*_LR_ *q* ^−^ at the apex (i.e., at $\dot{z}=0$ in the flight phase), where *B*_LR_ = diag(1, −1, −1, 1, −1, −1). In this study, *^+^ and *^−^ indicate the states immediately after and before reversing, respectively. Therefore, we focus on the touchdowns and liftoffs for only one pair of legs, specifically using the pair of legs FL and HR.

**FIGURE 2:**
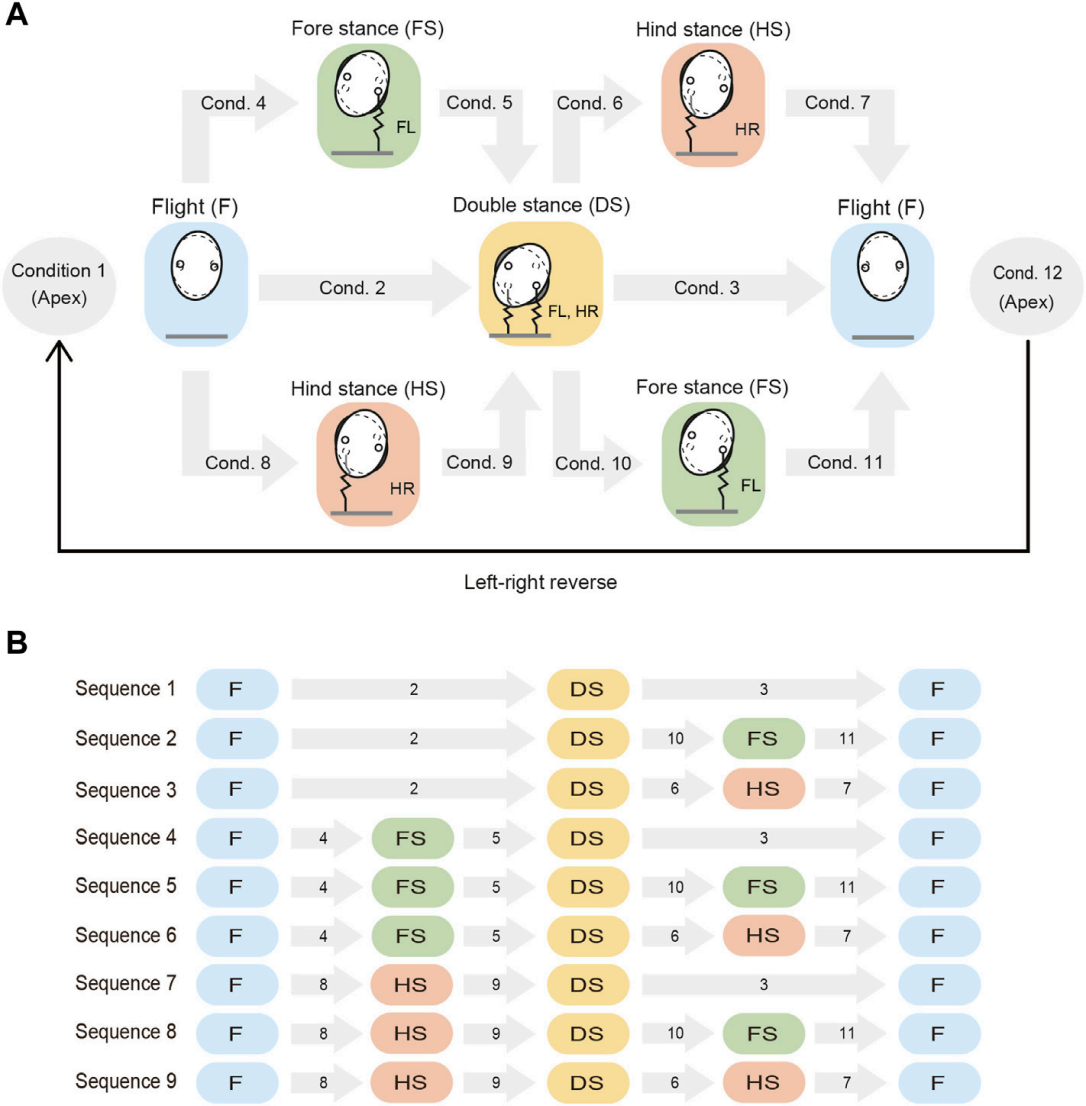
Phase transitions from an apex to the next apex. **(A)** Possible phases and the associated phase transitions. The phase transitions occur when conditions 1–12 are satisfied. The left and right sides of the model are then reversed at the next apex. **(B)** Nine sequences (Sequences 1–9) explain the phase transitions from an apex to the next apex. Each gray arrow with a number indicates the condition of the phase transition. Conditions 1 and 12 are not shown because they are common to all sequences.

The motion from one apex to the next apex is obtained from the phase transitions between the four phases (i.e., flight, fore stance, hind stance, and double stance phases), as illustrated in Figure 2. These phase transitions occur when the corresponding conditions (Conditions 1–12 in Figure 2) are satisfied. For example, the transition from the flight phase to the double stance phase occurs when Condition 2 is satisfied, when Conditions 4 and 5 are sequentially satisfied, or when Conditions 8 and 9 are sequentially satisfied. We use *r*_A_ = 0 to represent the condition where the COM reaches an apex, *r*_F_ = 0 to represent the condition where the fore leg of the pair touches and leaves the ground, *r*_H_ = 0 to represent the condition where the hind leg of the pair touches and leaves the ground, and *r*_D_ = 0 to represent the condition where both legs of the pair simultaneously touch and leave the ground. Specifically, Condition *i* (*i* = 1, … , 12) is given by

$$r_{i}\left(q\right)=\left\{\begin{array}{cc} r_{\text{A}}\left(q\right)=0\; & i=1,\;12\\ r_{\text{D}}\left(q\right)={\left\{r_{\text{F}}\left(q\right)\right\}}^{2}+{\left\{r_{\text{H}}\left(q\right)\right\}}^{2}=0\; & i=2,\;3\\ r_{\text{F}}\left(q\right)=0\; & i=4,\;6,\;9,\;11\\ r_{\text{H}}\left(q\right)=0\; & i=5,\;7,\;8,\;10, \end{array}\right.$$ (5)

where $r_{\text{A}}\left(q\right)=\dot{z}$ , *r*_F_(*q*) = *r*_FL_(*q*), and *r*_H_ = *r*_HR_(*q*).

Based on these phase transitions, the motion from one apex to the next apex can be explained using nine sequences (Sequences 1–9), as illustrated in Figure 2. In Sequence 1, both legs of the pair touch the ground simultaneously and then leave the ground simultaneously (flight–double stance–flight). In Sequence 2, both legs of the pair touch the ground simultaneously, but the hind leg then leaves the ground earlier than the fore leg (flight–double stance–fore stance–flight). In Sequence 3, both legs of the pair touch the ground simultaneously, but the foreleg then leaves the ground earlier than the hind leg (flight–double stance–hind stance–flight). In Sequence 4, the foreleg touches the ground earlier than the hind leg, but both legs then leave the ground simultaneously (flight–fore stance–double stance–flight). In Sequence 5, the foreleg touches the ground earlier than the hind leg, but the hind leg then leaves the ground earlier than the foreleg (flight–fore stance–double stance–fore stance–flight). In Sequence 6, the fore leg of the pair touches and then leaves the ground earlier than the hind leg (flight–fore stance–double stance–hind stance–flight). Sequences 7–9 are then obtained by exchanging the behavior of the fore and hind legs shown in Sequences 4–6, respectively.

### 2.3 Search of the Periodic Solutions and Stability Analysis

We search the periodic solutions using a Poincaré map by taking a Poincaré section immediately after the reversal of the left and right sides of the model at the apex $\left(\dot{z}=0\right)$ . Therefore, we define the state on the Poincaré section as $x={\left[z\;\theta_{\text{F}}\;\theta_{\text{H}}\;{\dot{\theta }}_{\text{F}}\;{\dot{\theta }}_{\text{H}}\right]}^{\text{T}}$ . The Poincaré map is then denoted by *x*_*i*+1_ = *P*(*x*_*i*_), where *x*_*i*_ is the state immediately after the reversal at the *i*th apex. A fixed point x* on the Poincaré section, which satisfies x* = *P*(x*), corresponds to a periodic solution. We search the periodic solutions numerically by solving the following:

$$S\left(x^{*}\right)=x^{*}-P\left(x^{*}\right)=0,$$ (6)

where we determine z* by comparing the simulation results and measured data of animals as described in Section 2.4.

We add a perturbation *δx*_*i*_ to the obtained solutions immediately after the reversal at the *i*th apex. The linearization of the Poincaré map *P* around x* yields

$$\delta x_{i+1}=J\delta x_{i},$$ (7)

where *J* is the Jacobian matrix of *P*. If all eigenvalues of *J* are located inside (inside and on) the unit circle on the complex plane, the periodic solution is asymptotically (marginally) stable; otherwise, the solution is unstable. Because the model is energy conservative, no asymptotically stable solutions exist. Therefore, we simply refer to marginally stable as stable. We define Λ = max_*i*=1,…,5_|*λ*_*i*_|, where *λ*_*i*_ (*i* = 1, … , 5) are the eigenvalues of *J*. If Λ = 1 is satisfied, the periodic solution is stable; otherwise, the solution is unstable.

### 2.4 Asymmetric Properties

Although we used the same physical parameters between the fore and hind bodies in the model in our previous study (Adachi et al., 2020), quadrupedal mammals, such as dogs and horses, generally have different physical properties between the fore and hind bodies. In particular, different body masses, moments of inertia, and leg stiffnesses greatly affect the locomotion dynamics. Because the difference of the mass between the fore and hind bodies has no effect on the equations of motion Eq. 3) of our model in the transverse plane (only the total mass has effects), we focus on the differences in the moments of inertia (*μ*_F_ and *μ*_H_) and leg stiffnesses (*k*_F_ and *k*_H_). To highlight the fore-aft asymmetry between these parameters, we define the averaged values of the moments of inertia and leg stiffnesses between the fore and hind bodies as *μ*_0_ and *k*_0_, respectively, and represent these four properties using asymmetric parameters *ɛ*_*μ*_ and *ɛ*_*k*_ as follows:

$$\mu_{\text{F}}=\left(1+\varepsilon_{\mu }\right)\mu_{0}$$ (8a)

$$\mu_{\text{H}}=\left(1-\varepsilon_{\mu }\right)\mu_{0}$$ (8b)

$$k_{\text{F}}=\left(1+\varepsilon_{k}\right)k_{0}$$ (8c)

$$k_{\text{H}}=\left(1-\varepsilon_{k}\right)k_{0}.$$ (8d)

*ɛ*_*μ*_ = *ɛ*_*k*_ = 0 corresponds to the symmetrical model used in our previous work (Adachi et al., 2020). Because the fore body of most quadrupedal mammals is typically heavier than the hind body (Rollinson and Martin, 1981) and the forelimbs support greater loads than the hind limbs (Merkens et al., 1993; Lee et al., 1999, 2004; Witte et al., 2004), we use *ɛ*_*μ*_, *ɛ*_*k*_ ≥ 0.

In this study, we use two types of physical parameter sets based on large breed dogs (e.g., German Shepherd), which have a short neck, and warmblood horses, which have a long neck. For dogs, we use *M*_F_ + *M*_H_ = 35 kg and *I*_F_ + *I*_H_ = 0.43 kgm^2^ based on Amit et al. (2009) and Jones et al. (2018) and *D* = 0.10 m based on the distance between the left and right hip joints of the hind limbs (Carrier et al., 2005; Belhaoues et al., 2020), which yields *μ*_0_ = 0.62. For horses, we use *M*_F_ + *M*_H_ = 538 kg and *I*_F_ + *I*_H_ = 37.5 kgm^2^ based on Buchner et al. (1997) and *D* = 0.22 m based on Gómez et al. (2009), which yields *μ*_0_ = 0.72. We also estimate *ɛ*_*μ*_ based on Buchner et al. (1997), Amit et al. (2009), and Jones et al. (2018) and *ɛ*_*k*_ based on Herr et al. (2002), which results in *ɛ*_*μ*_ = 0.12 and *ɛ*_*k*_ = 0.21 for dogs and *ɛ*_*μ*_ = 0.14 and *ɛ*_*k*_ = 0.25 for horses.

For the symmetric model (*ɛ*_*μ*_ = *ɛ*_*k*_ = 0) in our previous study (Adachi et al., 2020), the ratio of the leg and body-torsional spring constants, that is, *κ* = *k*_B_/*k*_0_, mainly determined the characteristics of the periodic solutions. In this study, we use *κ* instead of *k*_B_. We determine *k*_0_ and *κ* by comparing the simulation results and measured data of dogs and horses. Specifically, we first use the symmetric model (*ɛ*_*μ*_ = *ɛ*_*k*_ = 0, *μ*_0_ = 0.62 in the dog model and 0.72 in the horse model) to determine *k*_0_, *κ*, and z* so that the half cycle duration τ* (duration from an apex to the next apex), magnitude of the vertical movement *δ*_*z*_, and duty ratio averaged among the four legs *β*_0_ of the periodic solution minimize $V=c_{1}{\left(\tau^{*}-{\bar{\tau }}^{*}\right)}^{2}+c_{2}{\left(\delta_{z}-{\bar{\delta }}_{z}\right)}^{2}+c_{3}{\left(\beta_{0}-{\bar{\beta }}_{0}\right)}^{2}$ , where *c*_1_, *c*_2_, and *c*_3_ are the coefficients and ${\bar{\tau }}^{*}$ , ${\bar{\delta }}_{z}$ , and ${\bar{\beta }}_{0}$ are the measured data of τ*, *δ*_*z*_, and *β*_0_, respectively, during fast trotting in animals (Froude number is about 1.3; 3.5 m/s for dogs and 4.5 m/s for horses). In particular, we use ${\bar{\tau }}^{*}=1.9$ [0.2 s (Heglund et al., 1974; Maes et al., 2008)], ${\bar{\delta }}_{z}=0.11$ [0.011 m (Farley et al., 1993; Blickhan and Full, 1993)], and ${\bar{\beta }}_{0}=0.46$ (Fischer and Lilje, 2016; Maes et al., 2008) for the dog model and ${\bar{\tau }}^{*}=1.7$ [0.25 s (Heglund et al., 1974; Heglund and Taylor, 1988)], ${\bar{\delta }}_{z}=0.11$ [0.024 m (Blickhan and Full, 1993; Farley et al., 1993)], and ${\bar{\beta }}_{0}=0.4$ (Dutto et al., 2004; Bullimore and Burn, 2006) for the horse model. Because ${\bar{\tau }}^{*}$ is larger than ${\bar{\delta }}_{z}$ and ${\bar{\beta }}_{0}$ , we use *c*_1_ = 0.1 and *c*_2_ = *c*_3_ = 1. Using the obtained values of *k*_0_, *κ*, and z*, we then introduce asymmetry (*ɛ*_*k*_, *ɛ*_*μ*_) in the model (*ɛ*_*μ*_ = 0.12 and *ɛ*_*k*_ = 0.21 in dogs and *ɛ*_*μ*_ = 0.14 and *ɛ*_*k*_ = 2.5 in horses). Table 1 summarizes the parameters of the dog and horse models.

**TABLE 1:** Parameters of dog and horse models. *μ*_0_, *ɛ*_*μ*_, and *ɛ*_*k*_ are determined based on the measured data of animals and *k*_0_, *κ*, and z* are determined through the optimization of simulation.

Parameter	Value	
	**Dog**	**Horse**
*M*_F_ + *M*_H_ (kg)	35^a^	538^d^
*I*_F_ (kgm^2^)	0.26^a^	23.4^d^
*I*_H_ (kgm^2^)	0.17^a^	14.1^d^
*D* (m)	0.1^b^	0.22^e^
*K*_F_/*K*_H_	1.27^c^	1.33^c^
*μ*_0_	0.62	0.72
*ɛ*_*μ*_	0.12	0.14
*k*_0_	1.5	2.2
*ɛ*_*k*_	0.21	0.25
*κ*	0.20	0.21
z*	0.06	0.06

a: Amit et al. (2009); Jones et al. (2018), b: Carrier et al. (2005); Belhaoues et al. (2020), c: Herr et al. (2002), d: Buchner et al. (1997), e: Gómez et al. (2009).

## 3 Results

### 3.1 Effects of Asymmetry on the Gait Pattern

**FIGURE 3:**
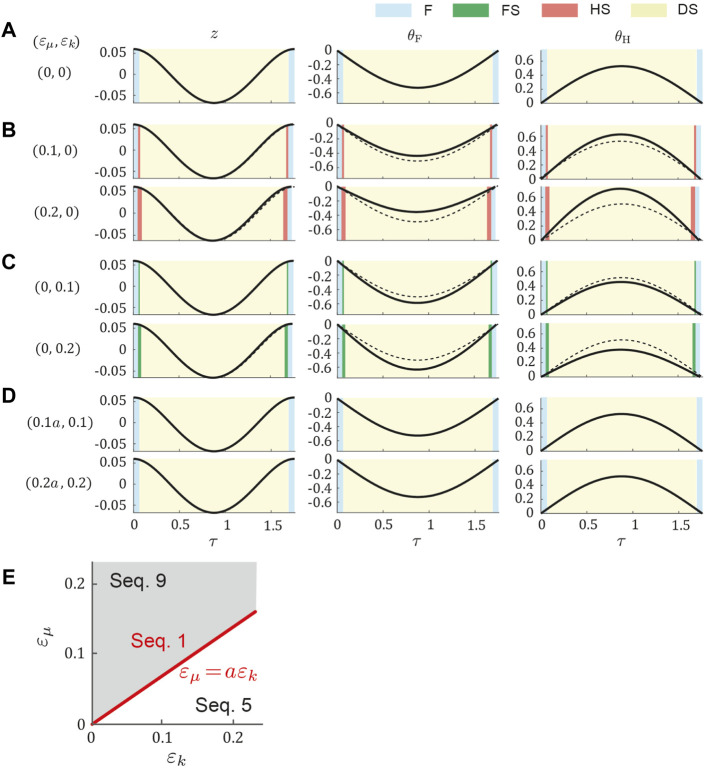
Gait dependence on *ɛ*_*k*_ and *ɛ*_*μ*_ in dog model. Time profile of periodic solution **(A)** for the symmetric model (*ɛ*_*k*_ = *ɛ*_*μ*_ = 0) and those for two values of **(B)** *ɛ*_*μ*_ with *ɛ*_*k*_ = 0, **(C)** *ɛ*_*k*_ with *ɛ*_*μ*_ = 0, and **(D)** *ɛ*_*k*_ with *ɛ*_*μ*_ = *aɛ*_*k*_. Cyan, green, pink, and yellow regions indicate flight (F), fore stance (FS), hind stance (HS), and double stance (DS), respectively. Dotted lines indicate the periodic solution of the symmetric model. **(E)** Gait dependence on *ɛ*_*k*_ and *ɛ*_*μ*_.

We obtained a periodic solution uniquely through the optimization in the symmetric model for dogs (*μ*_0_ = 0.62, *ɛ*_*μ*_ = *ɛ*_*k*_ = 0), which yielded *k*_0_ = 1.5, *κ* = 0.2, and z* = 0.06. By changing *ɛ*_*μ*_ and *ɛ*_*k*_ based on the symmetric periodic solution, we uniquely obtained the periodic solution for each set of (*ɛ*_*μ*_, *ɛ*_*k*_). Figure 3 shows the time profiles of typical periodic solutions. Regardless of *ɛ*_*μ*_ and *ɛ*_*k*_, the curve of *z* is sinusoidal and those of *θ*_F_ and *θ*_H_ are parabolic. When *ɛ*_*μ*_ = *ɛ*_*k*_ = 0, the magnitudes of *θ*_F_ and *θ*_H_ were identical and flight–double stance phase transition directly occurred (Figure 3A), which resulted in Sequence 1. When increasing *ɛ*_*μ*_ with *ɛ*_*k*_ = 0, the magnitude of *z* remained almost unchanged, whereas that of *θ*_F_ decreased and that of *θ*_H_ increased (Figure 3B). This made the stance phase durations of the hind legs longer than those of the forelegs and resulted in the appearance of the hind stance phase between the flight and double stance phases, which resulted in Sequence 9. By contrast, when we increased *ɛ*_*k*_ with *ɛ*_*μ*_ = 0, the magnitude of *θ*_F_ increased and that of *θ*_H_ decreased (Figure 3C), which is opposite to the result when we increased *ɛ*_*μ*_ in Figure 3B. This made the stance phase durations of the fore legs longer than those of the hind legs and resulted in the appearance of the fore stance phase between the flight and double stance phases, which means Sequence 5. Furthermore, we found a proportional relationship between *ɛ*_*μ*_ and *ɛ*_*k*_ (*ɛ*_*μ*_ = *aɛ*_*k*_, *a* = 0.69), which never changed the profiles of *θ*_F_ and *θ*_H_ from those in the symmetric model (*ɛ*_*μ*_ = *ɛ*_*k*_ = 0) and maintained Sequence 1 (Figure 3D). Sequence 5 appeared for *ɛ*_*μ*_ < *aɛ*_*k*_ and Sequence 9 appeared for *ɛ*_*μ*_ > *aɛ*_*k*_ (Figure 3E). The estimated values of the asymmetric parameters in the dog model (*ɛ*_*μ*_ = 0.12 and *ɛ*_*k*_ = 0.21) satisfied *ɛ*_*μ*_ < *aɛ*_*k*_ and thus generated Sequence 5. We compared the locomotion characteristics (half gait cycle duration, vertical displacement of COM, roll amplitude of the hind body, maximum vertical GRFs of the fore and hind legs, and duty ratios of the fore and hind legs) between simulation results using the estimated parameters of dogs and the measured data of dogs in Table 2. The locomotion characteristics of the simulation results are consistent with those of the measured data except for the roll amplitude of the hind body.

**TABLE 2:** Comparison of locomotion characteristics between models and animals using dimensionless values.

	Dog		Horse	
	Model	Animal	Model	Animal
Half cycle duration	1.75	1.9^a^	1.54	1.7^f^
Vertical COM displacement	0.12	0.11^b^	0.11	0.11^b^
Hind roll amplitude (deg)	29	6^c^	20	5^g^
Fore maximum GRF	1.1	1.5^d^	1.2	1.2^h^
Hind maximum GRF	0.7	0.8^d^	0.6	0.8^h^
Fore duty factor	0.47	0.48^e^	0.45	0.42^i^
Hind duty factor	0.46	0.44^e^	0.44	0.38^i^

a: Heglund et al. (1974); Maes et al. (2008), b: Farley et al. (1993); Blickhan and Full (1993), c: Fischer et al. (2018), d: Voss et al. (2010), e: Fischer and Lilje (2016); Maes et al. (2008), f: Heglund et al. (1974); Heglund and Taylor (1988), g: Byström et al. (2021), h: Merkens et al. (1993); Witte et al. (2004), i: Dutto et al. (2004); Bullimore and Burn (2006).

Similar to the dog model, we obtained a periodic solution and *k*_0_ = 2.2, *κ* = 0.21, and z* = 0.06 through the optimization in the symmetric model for horses (*μ*_0_ = 0.72, *ɛ*_*μ*_ = *ɛ*_*k*_ = 0). When changing *ɛ*_*μ*_ and *ɛ*_*k*_, we also achieved Sequences 1, 5, and 9 depending on *ɛ*_*μ*_⋚*aɛ*_*k*_, where *a* = 0.69 (see Supplementary Appendix SA). The estimated values of the asymmetric parameters in the horse model (*ɛ*_*μ*_ = 0.14 and *ɛ*_*k*_ = 0.25) also satisfied *ɛ*_*μ*_ < *aɛ*_*k*_ and generated Sequence 5. We compared the simulated locomotion characteristics using the estimated parameters of horses with the measured data of horses in Table 2. The locomotion characteristics of the simulation results of the horse model are also consistent with those of the measured data except for the roll amplitude of the hind body.

**FIGURE 4:**
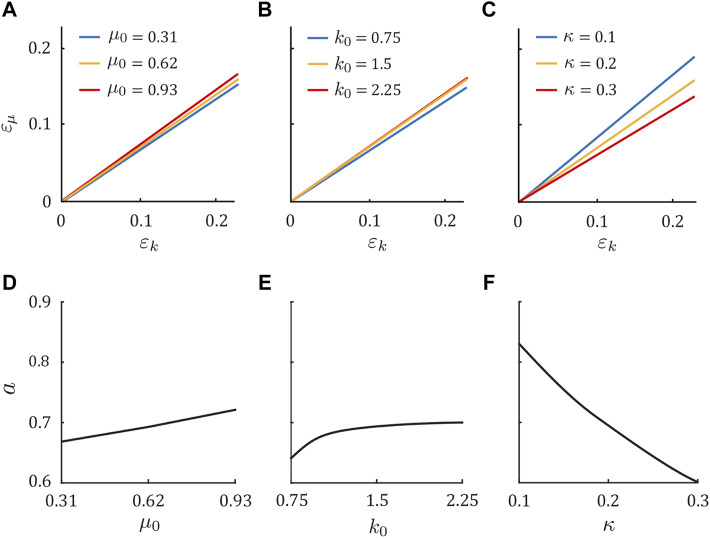
Gait dependence on physical parameters in the dog model. Condition of *ɛ*_*k*_ and *ɛ*_*μ*_ (*ɛ*_*μ*_ = *aɛ*_*k*_) to achieve Sequence 1 for three values of **(A)** *μ*_0_, **(B)** *k*_0_, and **(C)** *κ*, while holding the other parameters constant at *μ*_0_ = 0.62, *k*_0_ = 1.5, and *κ* = 0.2. Sequences 5 and 9 appeared when *ɛ*_*μ*_ < *aɛ*_*k*_ and *ɛ*_*μ*_ > *aɛ*_*k*_, respectively. Dependence of *a* on **(D)** *μ*_0_, **(E)** *k*_0_, and **(F)** *κ*.

Next, we investigated the phase transition of the periodic solution by independently changing *μ*_0_, *k*_0_, and *κ* by ± 50*%* from the dog parameter set (*μ*_0_ = 0.62, *k*_0_ = 1.5, and *κ* = 0.2) in Figure 4. In the same manner as the above results, Sequences 1, 5, and 9 appeared for *ɛ*_*μ*_ = *aɛ*_*k*_, *ɛ*_*μ*_ < *aɛ*_*k*_, and *ɛ*_*μ*_ > *aɛ*_*k*_, respectively. Although the coefficient *a* changed slightly when *μ*_0_ and *k*_0_ increased (Figures 4A,B,D,E), it largely decreased as *κ* increased (Figures 4C,F). These tendencies were also observed in the horse model (see Supplementary Appendix SB).

### 3.2 Effects of Asymmetry on Gait Stability

**FIGURE 5:**
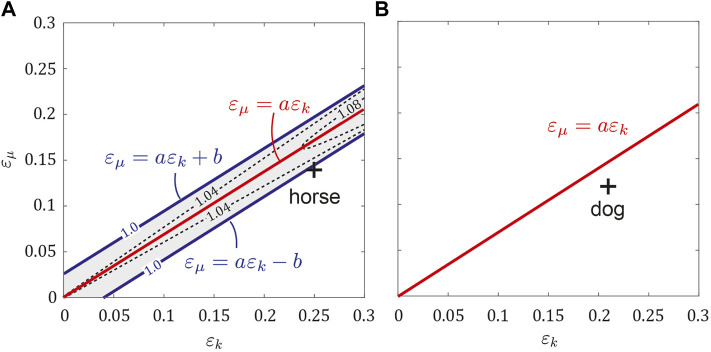
Stability of periodic solutions for *ɛ*_*μ*_ and *ɛ*_*k*_. Contour of the maximum eigenvalue Λ in the **(A)** horse model and **(B)** dog model. White and gray regions indicate the stable and unstable regions, respectively. *ɛ*_*μ*_ = *aɛ*_*k*_ ± *b* corresponds to the boundary of Λ = 1. *b* > 0 in the horse model, whereas *b* = 0 in the dog model. Crosses indicate the estimated values of *ɛ*_*μ*_ and *ɛ*_*k*_ in animals.

We investigated the stability of the obtained periodic solutions for *ɛ*_*k*_ and *ɛ*_*μ*_ using the horse parameter set (*μ*_0_, *k*_0_, *κ*) = (0.72, 2.2, 0.21) by calculating the maximum eigenvalue Λ of the Jacobian matrix of the Poincaré map (Figure 5A). When *ɛ*_*μ*_ = *aɛ*_*k*_ was satisfied (including *ɛ*_*k*_ = *ɛ*_*μ*_ = 0), Λ was much larger than 1 and the periodic solutions were highly unstable (the instability increased as *ɛ*_*k*_ increased). As the distance of (*ɛ*_*k*_, *ɛ*_*μ*_) from *ɛ*_*μ*_ = *aɛ*_*k*_ increased, Λ decreased. The solutions became stable with Λ = 1 when (*ɛ*_*k*_, *ɛ*_*μ*_) moved across two parallel lines *ɛ*_*μ*_ = *aɛ*_*k*_ ± *b*, where *b* = 0.02. This means that the trotting of the symmetric horse model was unstable, whereas fore-aft asymmetry stabilized it. The estimated asymmetric parameters of horses (*ɛ*_*μ*_ = 0.14 and *ɛ*_*k*_ = 0.25) satisfied *ɛ*_*μ*_ < *aɛ*_*k*_ − *b*, which indicates that the trotting of horses was stable. By contrast, the periodic solutions for the dog parameter set (*μ*_0_, *k*_0_, *κ*) = (0.62, 1.5, 0.2) were always stable for 0 ≤ *ɛ*_*μ*_ ≤ 0.3 and 0 ≤ *ɛ*_*k*_ ≤ 0.3, including the symmetric case *ɛ*_*k*_ = *ɛ*_*μ*_ = 0 (Figure 5B). This result corresponds to *b* = 0 in the above horse model. Therefore, trotting of dogs was also stable with respect to the estimated asymmetric parameters of dogs (*ɛ*_*μ*_ = 0.12 and *ɛ*_*k*_ = 0.21).

**FIGURE 6:**
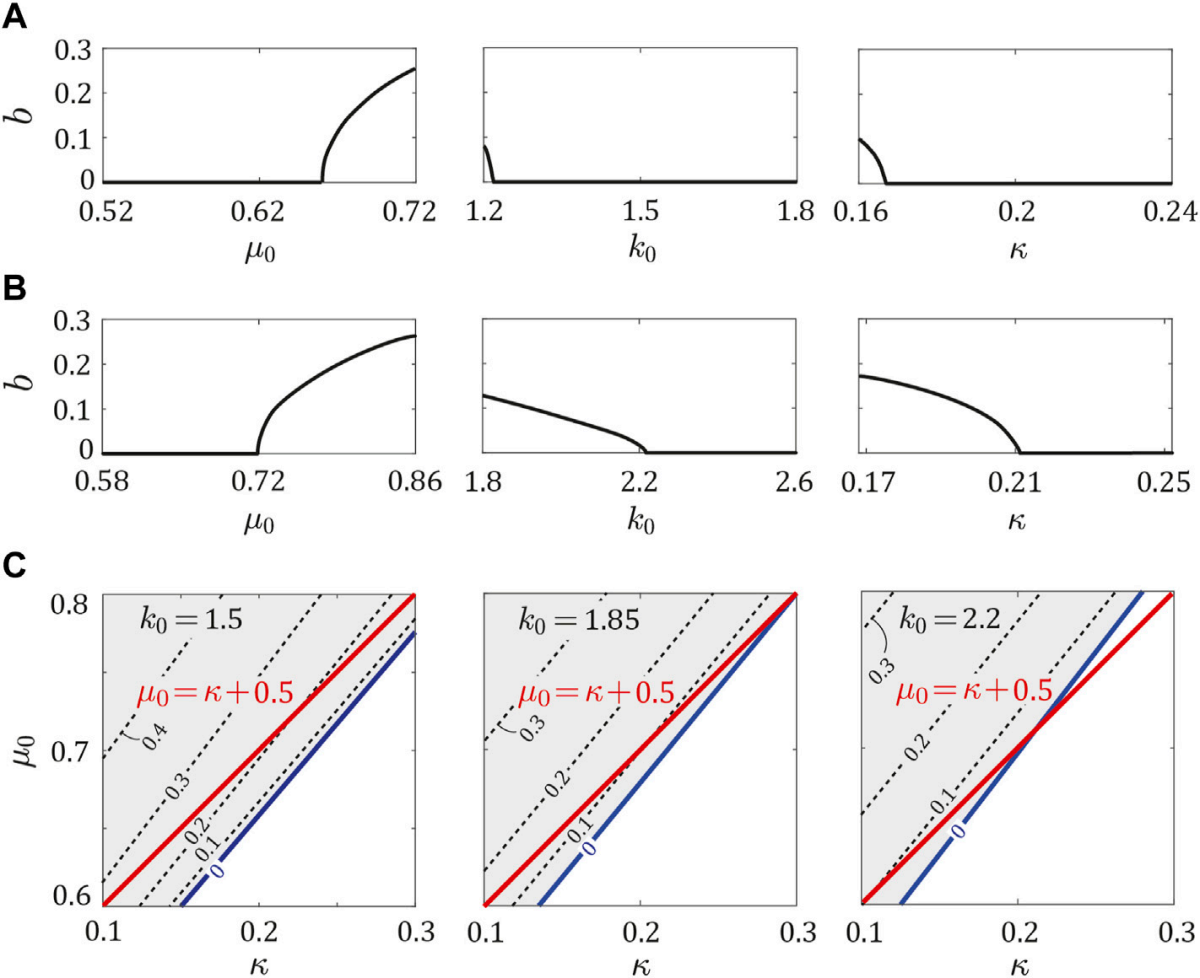
Dependence of *b* on *μ*_0_, *k*_0_, and, *κ*. *b* vs., *μ*_0_, *k*_0_, and, *κ* using the parameter sets for **(A)** dogs and **(B)** horses, while holding the other parameters constant at (*μ*_0_, *k*_0_, *κ*) = (0.62, 1.5, 0.2) for dogs and (0.72, 2.2, 0.21) for horses. **(C)** Contour of *b* around *μ*_0_ = *κ* + 0.5 for *k*_0_ = 1.5, 1.85, and 2.2. Red and blue lines indicate *μ*_0_ = *κ* + 0.5 and boundary of *b* = 0 and *b* > 0, respectively. White and gray regions indicate *b* = 0 and *b* > 0, respectively.

Next, we investigated the stability of the periodic solutions in the asymmetric model (*ɛ*_*k*_, *ɛ*_*μ*_ ≥ 0) by independently changing *μ*_0_, *k*_0_, and *κ* from the parameter sets of dogs and horses. We found that *ɛ*_*μ*_ = *aɛ*_*k*_ ± *b* determined the stability for both cases in the same manner as that for the above results. Specifically, if *b* = 0, the periodic solutions were stable regardless of *ɛ*_*μ*_ and *ɛ*_*k*_. By contrast, if *b* > 0, while the periodic solutions were unstable when *ɛ*_*μ*_ = *ɛ*_*k*_ = 0, they became stable when *ɛ*_*μ*_ ≥ *aɛ*_*k*_ + *b* or *ɛ*_*μ*_ ≤ *aɛ*_*k*_ − *b*. Therefore, although large asymmetry was necessary as *b* increased, fore-aft asymmetry stabilized the periodic solutions. We examined whether *b* depended on *μ*_0_, *k*_0_, and *κ* in the same manner as *a* in Figure 4. Specifically, we investigated *b* by independently changing *μ*_0_, *k*_0_, and *κ* by ± 20*%* from the parameter sets for dogs (*μ*_0_ = 0.62, *k*_0_ = 1.5, and *κ* = 0.2) and horses (*μ*_0_ = 0.72, *k*_0_ = 2.2, and *κ* = 0.21) in Figures 6A,B, respectively. In both parameter sets for dogs and horses, when *μ*_0_ exceeded a certain value, *b* increased from 0. When *k*_0_ or *κ* fell below a certain value, *b* increased from 0.

In our previous study (Adachi et al., 2020) using the symmetric model (*ɛ*_*μ*_ = *ɛ*_*k*_ = 0), we demonstrated that *k*_0_ hardly affected the stability of the periodic solutions, and *μ*_0_ and *κ* mainly determined the stability. Specifically, the periodic solutions were stable when *μ*_0_ ≤ *κ* + 0.5 and unstable when *μ*_0_ > *κ* + 0.5. We investigated *b* around this stability boundary (*μ*_0_ = *κ* + 0.5) with *k*_0_ = 1.5, 1.85, and 2.2 in Figure 6C, where *k*_0_ = 1.5 and *k*_0_ = 2.2 correspond to the dog and horse parameters, respectively. For each value of *k*_0_, the boundary between *b* = 0 and *b* > 0 existed around *μ*_0_ = *κ* + 0.5.

## 4 Discussion

### 4.1 Effects of Fore-Aft Asymmetry on the Transverse Dynamics of Trotting

Regardless of the dog and horse models, we found periodic solutions, which had several types of phase transitions depending on the asymmetric parameters *ɛ*_*μ*_ and *ɛ*_*k*_. Specifically, Sequences 1 (flight–double stance–flight), 5 (flight–fore stance–double stance–fore stance–flight), and 9 (flight–hind stance–double stance–hind stance–flight) appeared when *ɛ*_*μ*_ = *aɛ*_*k*_, *ɛ*_*μ*_ < *aɛ*_*k*_, and *ɛ*_*μ*_ > *aɛ*_*k*_, respectively (Figure 3), where *a* depended on the physical parameter set (*μ*_0_, *k*_0_, and *κ*) as shown in Figure 4. For us to understand the mechanism for generating these sequences, understanding the relationship between the averaged angle of the fore and hind bodies (*θ* = (*θ*_F_ + *θ*_H_)/2) and torsional angle (*ϕ* = (*θ*_F_ − *θ*_H_)/2) is crucial. Sequence 1 requires simultaneous touchdowns and liftoffs by the paired fore and hind legs. In our previous study (Adachi et al., 2020) using the symmetric model (*ɛ*_*μ*_ = *ɛ*_*k*_ = 0), we demonstrated that Sequence 1 appeared only when *θ* = 0, that is, the fore and hind bodies always rotated in the opposite direction (*θ*_F_ = −*θ*_H_). In the present study, we demonstrated that even if the model had asymmetric properties *ɛ*_*μ*_ and *ɛ*_*k*_, the relationship *ɛ*_*μ*_ = *aɛ*_*k*_ produced *θ*_F_ = −*θ*_H_ (*θ* = 0), which resulted in Sequence 1. This relationship was analytically obtained using perturbation theory (see Supplementary Appendix SC). By contrast, because *ɛ*_*μ*_ ≠ *aɛ*_*k*_ caused *θ*_F_ ≠ −*θ*_H_ (*θ* ≠ 0), other sequences appeared. In particular, when *θ* and *ϕ* had the same sign, the rotation of the fore body (*θ*_F_ = *θ* + *ϕ*) became larger than that of the hind body (*θ*_H_ = *θ* − *ϕ*), which induced Sequence 5. When *θ* and *ϕ* had opposite signs, the rotation of the hind body became larger than that of the fore body, which induced Sequence 9.

In previous studies (Zou and Schmiedeler 2006; Yamada et al., 2022), the researchers used a single rigid body for their simple models to investigate quadrupedal bounding in the sagittal plane and demonstrated that the fore-aft asymmetry of the CoM position of the body reduced gait stability. However, our results demonstrate that even if the trotting of the fore-aft symmetric model with *ɛ*_*μ*_ = *ɛ*_*k*_ = 0 in the transverse plane was unstable, it was stabilized by introducing *ɛ*_*μ*_ and *ɛ*_*k*_ to satisfy *ɛ*_*μ*_ < *aɛ*_*k*_ − *b* or *ɛ*_*μ*_ > *aɛ*_*k*_ + *b* (Figure 5), where *b* also depended on the physical parameter set (*μ*_0_, *k*_0_, and *κ*) as shown in Figure 6; that is, fore-aft asymmetry did not reduce gait stability, but rather improved it in the transverse plane. These different effects of asymmetry on gait stability were mainly caused by different effects on the entire dynamics. Specifically, because in previous studies (Zou and Schmiedeler, 2006; Yamada et al., 2022), researchers used a single rigid body in the model and incorporated fore-aft asymmetry in the single body, the asymmetry directly affected the entire dynamics. By contrast, we used two segmented bodies in our model and incorporated fore-aft asymmetries as different properties between the bodies. The fore-aft asymmetries indirectly affected the entire dynamics via the torsional body joint that connected the two bodies.

In this study, the boundary between *b* = 0 and *b* > 0 existed near *μ*_0_ = *κ* + 0.5 in the *μ*_0_-*κ* plane (Figure 6), which corresponds to the stability boundary (*μ*_0_ ≤ *κ* + 0.5: stable, *μ*_0_ > *κ* + 0.5: unstable) in the symmetric model (*ɛ*_*k*_ = *ɛ*_*μ*_ = 0), as achieved in (Adachi et al., 2020). When *μ*_0_ < *κ* + 0.5, the introduction of *ɛ*_*μ*_ and *ɛ*_*k*_ to the symmetric model never changed the stability, and the periodic solutions remained stable, which resulted in *b* = 0 for the stability condition *ɛ*_*μ*_ ≷ *aɛ*_*k*_ ± *b* in Figure 5. By contrast, when *μ*_0_ > *κ* + 0.5, the introduction of *ɛ*_*μ*_ and *ɛ*_*k*_ made the periodic solutions stable for *ɛ*_*μ*_ ≷ *aɛ*_*k*_ ± *b* (*b* > 0). Therefore, we expect that the boundary between *b* = 0 and *b* > 0 is identical to *μ*_0_ = *κ* + 0.5 in the *μ*_0_-*κ* plane. However, our results had some differences between them, as shown in Figure 6. This is mainly because we obtained the periodic solutions numerically based on the non-linear governing equations, whereas in our previous work (Adachi et al., 2020), we obtained them approximately by linearizing the governing equations.

### 4.2 Biological Relevance of Our Findings

Our results showed that the fore-aft asymmetry improves gait stability during trotting. Although our model incorporated only passive forces using springs, unstable gait can be stabilized by additional control inputs. However, when the system has passive stability, it needs less control inputs and sensory feedbacks. This results in low energy consumption, which is therefore beneficial for quadrupedal animals.

Because the front part of the body is generally heavier and has larger moment of inertia in the transverse plane than the hind part in quadrupeds (Rollinson and Martin, 1981), the forelegs need to generate more impulse than the hind legs to achieve trotting by inhibiting body pitching. In fact, the stance phase durations of the fore legs are basically longer than those of the hind legs during trotting in quadrupeds (Merkens et al., 1993; Lee et al., 1999; Weishaupt et al., 2004; Robilliard et al., 2007; Fischer and Lilje, 2016). These characteristics appeared only in the periodic solutions with Sequence 5 in our model.

In this study, we used the parameter set estimated in dogs. The average and difference of the moments of inertia between the fore and hind bodies were both relatively small (*μ*_0_ = 0.62 and *ɛ*_*μ*_ = 0.12) and *μ*_0_ < *κ* + 0.5 was satisfied (i.e., the periodic solution of the symmetric model was stable). As a result, we achieved *b* = 0 and the periodic solutions were stable even when we introduced the asymmetries *ɛ*_*μ*_ and *ɛ*_*k*_. Additionally, *ɛ*_*μ*_ < *aɛ*_*k*_ was satisfied for the estimated values in dogs, which yielded Sequence 5 (Figure 5B). These characteristics are consistent with those of trotting in dogs (Lee et al., 1999, 2004; Lee, 2010; Fischer and Lilje, 2016). By contrast, horses have a longer neck than dogs (Loscher et al., 2016) and the estimated average and difference of the moments of inertia were both larger than those of dogs (*μ*_0_ = 0.72 and *ɛ*_*μ*_ = 0.14). The periodic solution of the symmetric horse model with *ɛ*_*μ*_ = *ɛ*_*k*_ = 0 was unstable. However, that was stabilized by making the fore legs stiffer (*ɛ*_*k*_ = 0.25, Figure 5A). Researchers have suggested that horses enhance the elasticity of their fore legs using their thoracic muscles, such as serratus ventralis thoracis, and generate a large difference in stiffness between their fore and hind legs (Payne et al., 2005b).

### 4.3 Limitations of Our Study and Future Work

In this study, we investigated the effects of fore-aft asymmetry on quadrupedal trotting in the transverse plane using a simple model. Our results demonstrated that asymmetry improves gait stability. In addition, many locomotion characteristics of the simulation results were consistent with those of the measured data of animals, as shown in Table 2. However, our model does not necessarily explain all phenomena of trotting in animals and has limitations. For example, the roll amplitude of the hind body in our models was larger than that of the measured data of animals (Table 2). This discrepancy could be due to the different joint structure at the leg roots. Specifically, although we used smooth rotational joints, quadrupeds have muscles around the joints, which prevent large leg abduction (Schilling et al., 2009). In the future, we would like to incorporate this effect of the muscles around the leg roots to our model.

Secondary, our horse model showed Sequence 5 (flight–fore stance–double stance–fore stance–flight), where the fore leg of the pair touches the ground earlier and leaves it later than the hind leg. Although horses show Sequence 5 during trotting (Weishaupt et al., 2004), they basically show Sequence 6 (flight–fore stance–double stance–hind stance–flight), where the fore leg of the pair touches and leaves the ground earlier than the hind leg (Hildebrand, 1965). One possible reason for this discrepancy is the absence of pitching dynamics in our model. Lee (Lee, 2010) demonstrated that the disturbance of trotting in dogs that results from changing the ground inclination and added mass position changes their foot pattern through the regulation of balance in pitching. Additionally, quadrupeds whose COM is located in an extremely forward position (which corresponds to large *ɛ*_*μ*_ in this study), such as gnus, do not use trotting, but do use walking and cantering (Pennycuick, 1975). This is mainly because it is difficult for them to keep the body pitching parallel to the ground with the extreme fore-aft asymmetry. To investigate these characteristics in quadrupedal locomotion, we would like to introduce pitching dynamics in our model in the future.

## 5 Conclusion

In this study, we examined the effects of fore-aft asymmetry on trotting by quadrupedal mammals in the transverse plane using a simple model. Our results demonstrated that the asymmetry gives different foot patterns and improves gait stability. Our findings improve our understanding of gait dynamics in quadrupeds with fore-aft asymmetry.

## Data Availability

The raw data supporting the conclusion of this article will be made available by the authors, without undue reservation.

## References

[B1] Adachi M. Aoi S. Kamimura T. Tsuchiya K. Matsuno F. (2020). Body Torsional Flexibility Effects on Stability during Trotting and Pacing Based on a Simple Analytical Model. Bioinspir. Biomim. 15, 055001. 10.1088/1748-3190/ab968d 32454464

[B2] Alexander R. M. Jayes A. S. (1983). A Dynamic Similarity Hypothesis for the Gaits of Quadrupedal Mammals. J. Zoolog. 201, 135–152. 10.1111/j.1469-7998.1983.tb04266.x

[B3] Amit T. Gomberg B. R. Milgram J. Shahar R. (2009). Segmental Inertial Properties in Dogs Determined by Magnetic Resonance Imaging. Vet. J. 182, 94–99. 10.1016/j.tvjl.2008.05.024 18691919

[B4] Belhaoues F. Breit S. Forstenpointner G. Gardeisen A. (2020). Sexual Dimorphism in Limb Long Bones of the German shepherd Dog. Anat. Histol. Embryol. 49, 464–477. 10.1111/ahe.12550 32157727

[B5] Berkemeier M. D. (1998). Modeling the Dynamics of Quadrupedal Running. Int. J. Robotics Res. 17, 971–985. 10.1177/027836499801700905

[B6] Bertram J. E. A. Gutmann A. (2008). Motions of the Running Horse and Cheetah Revisited: Fundamental Mechanics of the Transverse and Rotary Gallop. J. R. Soc. Interf. 6, 549–559. 10.1098/rsif.2008.0328 PMC2696142 18854295

[B7] Blickhan R. Full R. J. (1993). Similarity in Multilegged Locomotion - Bouncing like a Monopode. J. Comp. Physiol. 173, 509–517. 10.1007/bf00197760

[B8] Buchner H. H. F. Savelberg H. H. C. M. Schamhardt H. C. Barneveld A. (1997). Inertial Properties of Dutch Warmblood Horses. J. Biomech. 30, 653–658. 10.1016/s0021-9290(97)00005-5 9165402

[B9] Bullimore S. R. Burn J. F. (2006). Dynamically Similar Locomotion in Horses. J. Exp. Biol. 209, 455–465. 10.1242/jeb.02029 16424095

[B10] Byström A. Hardeman A. M. Serra Bragança F. M. Roepstorff L. Swagemakers J. H. van Weeren P. R. (2021). Differences in Equine Spinal Kinematics between Straight Line and circle in Trot. Sci. Rep. 11, 12832. 10.1038/s41598-021-92272-2 34145339 PMC8213771

[B11] Carrier D. R. Chase K. Lark K. G. (2005). Genetics of Canid Skeletal Variation: Size and Shape of the Pelvis. Genome Res. 15, 1825–1830. 10.1101/gr.3800005 16339381 PMC1356121

[B12] De A. Koditschek D. E. (2018). Vertical Hopper Compositions for Preflexive and Feedback-Stabilized Quadrupedal Bounding, Pacing, Pronking, and Trotting. Int. J. Robotics Res. 37, 743–778. 10.1177/0278364918779874

[B13] Dunbar D. C. Macpherson J. M. Simmons R. W. Zarcades A. (2008). Stabilization and Mobility of the Head, Neck and Trunk in Horses during Overground Locomotion: Comparisons with Humans and Other Primates. J. Exp. Biol. 211, 3889–3907. 10.1242/jeb.020578 19043061 PMC2768006

[B14] Dutto D. J. Hoyt D. F. Cogger E. A. Wickler S. J. (2004). Ground Reaction Forces in Horses Trotting up an Incline and on the Level over a Range of Speeds. J. Exp. Biol. 207, 3507–3514. 10.1242/jeb.01171 15339946

[B15] Farley C. T. Glasheen J. McMahon T. A. (1993). Running Springs: Speed and Animal Size. J. Exp. Biol. 185, 71–86. 10.1242/jeb.185.1.71 8294853

[B16] Fischer M. S. Lehmann S. V. Andrada E. (2018). Three-dimensional Kinematics of Canine Hind Limbs: *In Vivo*, Biplanar, High-Frequency Fluoroscopic Analysis of Four Breeds during Walking and Trotting. Sci. Rep. 8, 16982. 10.1038/s41598-018-34310-0 30451855 PMC6242825

[B17] Fischer M. S. Lilje K. E. (2016). Dogs in Motion. Cowbridge, United Kingdom: The Pet Book Publishing Company Ltd.

[B18] Full R. J. Koditschek D. E. (1999). Templates and Anchors: Neuromechanical Hypotheses of Legged Locomotion on Land. J. Exp. Biol. 202, 3325–3332. 10.1242/jeb.202.23.3325 10562515

[B19] Gillette R. L. Angle T. C. (2008). Recent Developments in Canine Locomotor Analysis: a Review. Vet. J. 178, 165–176. 10.1016/j.tvjl.2008.01.009 18406641

[B20] Gómez M. D. Valera M. Molina A. Gutiérrez J. P. Goyache F. (2009). Assessment of Inbreeding Depression for Body Measurements in Spanish Purebred (Andalusian) Horses. Livestock Sci. 122, 149–155. 10.1016/j.livsci.2008.08.007

[B21] Heglund N. C. Taylor C. R. McMahon T. A. (1974). Scaling Stride Frequency and Gait to Animal Size: Mice to Horses. Science 186, 1112–1113. 10.1126/science.186.4169.1112 4469699

[B22] Heglund N. C. Taylor C. R. (1988). Speed, Stride Frequency and Energy Cost Per Stride: How Do They Change with Body Size and Gait? J. Exp. Biol. 138, 301–318. 10.1242/jeb.138.1.301 3193059

[B23] Herr H. M. Huang G. T. McMahon T. A. (2002). A Model of Scale Effects in Mammalian Quadrupedal Running. J. Exp. Biol. 205, 959–967. 10.1242/jeb.205.7.959 11916991

[B24] Hildebrand M. Goslow G. E. J. (2001). Analysis of Vertebrate Structure. 5th edition. New York: Wiley.

[B25] Hildebrand M. (1968). Symmetrical Gaits of Dogs in Relation to Body Build. J. Morphol. 124, 353–359. 10.1002/jmor.1051240308 5657937

[B26] Hildebrand M. (1965). Symmetrical Gaits of Horses. Science 150, 701–708. 10.1126/science.150.3697.701 5844074

[B27] Jones O. Y. Raschke S. U. Riches P. E. (2018). Inertial Properties of the German Shepherd Dog. PLOS ONE 13, e0206037. 10.1371/journal.pone.0206037\\} 30339688 PMC6195294

[B28] Kilbourne B. M. Hoffman L. C. (2013). Scale Effects between Body Size and Limb Design in Quadrupedal Mammals. PLOS ONE 8, e78392. 10.1371/journal.pone.0078392 24260117 PMC3832634

[B29] Lee D. V. Bertram J. E. Todhunter R. J. (1999). Acceleration and Balance in Trotting Dogs. J. Exp. Biol. 202, 3565–3573. 10.1242/jeb.202.24.3565 10574733

[B30] Lee D. V. Stakebake E. F. Walter R. M. Carrier D. R. (2004). Effects of Mass Distribution on the Mechanics of Level Trotting in Dogs. J. Exp. Biol. 207, 1715–1728. 10.1242/jeb.00947 15073204

[B31] Lee J. Y. Lee S. J. (2010). Hemodynamics of the Omphalo-Mesenteric Arteries in Stage 18 Chicken Embryos and "Flow-Structure" Relations for the Microcirculation. Microvasc. Res. 80, 402–411. 10.1016/j.mvr.2010.08.003 20727902

[B32] Loscher D. M. Meyer F. Kracht K. Nyakatura J. A. (2016). Timing of Head Movements Is Consistent with Energy Minimization in Walking Ungulates. Proc. R. Soc. B. 283, 20161908. 10.1098/rspb.2016.1908 PMC5136594 27903873

[B33] Maes L. D. Herbin M. Hackert R. Bels V. L. Abourachid A. (2008). Steady Locomotion in Dogs: Temporal and Associated Spatial Coordination Patterns and the Effect of Speed. J. Exp. Biol. 211, 138–149. 10.1242/jeb.008243 18083742

[B34] Merkens H. W. Schamhardt H. C. Osch G. J. V. M. Bogert A. J. (1993). Ground Reaction Force Patterns of Dutch Warmblood Horses at normal Trot. Equine Vet. J. 25, 134–137. 10.1111/j.2042-3306.1993.tb02923.x 8467772

[B35] Muybridge E. (1957). Animals in Motion. New York: Dover Publications.

[B36] Payne R. C. Hutchinson J. R. Robilliard J. J. Smith N. C. Wilson A. M. (2005a). Functional Specialisation of Pelvic Limb Anatomy in Horses (*Equus caballus*). J. Anat. 206, 557–574. 10.1111/j.1469-7580.2005.00420.x 15960766 PMC1571521

[B37] Payne R. C. Veenman P. Wilson A. M. (2005b). The Role of the Extrinsic Thoracic Limb Muscles in Equine Locomotion. J. Anat. 206, 193–204. 10.1111/j.1469-7580.2005.00353.x 15730484 PMC1571467

[B38] Pennycuick C. J. (1975). On the Running of the Gnu (*Connochaetes taurinus*) and Other Animals. J. Exp. Biol. 63, 775–799. 10.1242/jeb.63.3.775

[B39] Robilliard J. J. Pfau T. Wilson A. M. (2007). Gait Characterisation and Classification in Horses. J. Exp. Biol. 210, 187–197. 10.1242/jeb.02611 17210956

[B40] Rollinson J. Martin R. D. (1981). Comparative Aspects of Primate Locomotion with Special Reference to Arboreal Cercopithecines. Symp. Zool. Soc. Lond. 48, 377–427.

[B41] Schilling N. Carrier D. R. (2009). Function of the Epaxial Muscles during Trotting. J. Exp. Biol. 212, 1053–1063. 10.1242/jeb.020248 19282502

[B42] Schilling N. Fischbein T. Yang E. P. Carrier D. R. (2009). Function of the Extrinsic Hindlimb Muscles in Trotting Dogs. J. Exp. Biol. 212, 1036–1052. 10.1242/jeb.020255 19282501

[B43] Voss K. Galeandro L. Wiestner T. Haessig M. Montavon P. M. (2010). Relationships of Body Weight, Body Size, Subject Velocity, and Vertical Ground Reaction Forces in Trotting Dogs. Vet. Surg. 39, 863–869. 10.1111/j.1532-950x.2010.00729.x 20825596

[B44] Weishaupt M. A. Wiestner T. Hogg H. P. Jordan P. Auer J. A. (2004). Vertical Ground Reaction Force-Time Histories of Sound Warmblood Horses Trotting on a Treadmill. Vet. J. 168, 304–311. 10.1016/j.tvjl.2003.08.007 15501148

[B45] Williams S. B. Wilson A. M. Daynes J. Peckham K. Payne R. C. (2008a). Functional Anatomy and Muscle Moment Arms of the Thoracic Limb of an Elite Sprinting Athlete: the Racing Greyhound (*Canis familiaris*). J. Anat. 213, 373–382. 10.1111/j.1469-7580.2008.00962.x 19034998 PMC2644772

[B46] Williams S. B. Wilson A. M. Rhodes L. Andrews J. Payne R. C. (2008b). Functional Anatomy and Muscle Moment Arms of the Pelvic Limb of an Elite Sprinting Athlete: the Racing Greyhound (*Canis familiaris*). J. Anat. 213, 361–372. 10.1111/j.1469-7580.2008.00961.x 18657259 PMC2644771

[B47] Witte T. H. Knill K. Wilson A. M. (2004). Determination of Peak Vertical Ground Reaction Force from Duty Factor in the Horse (*Equus caballus*). J. Exp. Biol. 207, 3639–3648. 10.1242/jeb.01182 15371472

[B48] Yamada T. Aoi S. Adachi M. Kamimura T. Higurashi Y. Wada N. (2022). Center of Mass Offset Enhances the Selection of Transverse Gallop in High-Speed Running by Horses: a Modeling Study. Front. Bioeng. Biotechnol. 10, 825157. 10.3389/fbioe.2022.825157 35295643 PMC8919080

[B49] Zou H. Schmiedeler J. P. (2006). The Effect of Asymmetrical Body-Mass Distribution on the Stability and Dynamics of Quadruped Bounding. IEEE Trans. Robot. 22, 711–723. 10.1109/tro.2006.875477

