# Supplementary material for: Fore-Aft Asymmetry Improves the Stability of Trotting in the Transverse Plane: A Modeling Study
Source: Front Bioeng Biotechnol. 2022 Jun 3;10:807777. doi: 10.3389/fbioe.2022.807777 (PMC9203715; doi:10.3389/fbioe.2022.807777)
Supplement: Supplementary file 1 [file DataSheet1.pdf]

Supplementary Material

A PERIODIC SOLUTIONS IN THE HORSE MODEL

Figure S1 shows the time profiles of typical periodic solutions obtained by changing ε_μ and ε_k from the symmetric horse model ($\varepsilon_\mu = \varepsilon_k = 0$, $\mu_0 = 0.72$, $k_0 = 2.2$, $\kappa = 0.21$, and $z^* = 0.06$). When $\varepsilon_\mu = \varepsilon_k = 0$, Sequence 1 appeared (Figure S1A). When increasing ε_μ with $\varepsilon_k = 0$, Sequence 9 appeared (Figure S1B). By contrast, when increasing ε_k with $\varepsilon_\mu = 0$, Sequence 5 appeared (Figure S1C). Furthermore, when $\varepsilon_\mu = a\varepsilon_k$ ($a = 0.69$), Sequence 1 appeared in the same manner as the symmetric model (Figure S1D). Sequence 5 appeared for $\varepsilon_\mu < a\varepsilon_k$ and Sequence 9 appeared for $\varepsilon_\mu > a\varepsilon_k$ (Figure S1E). These characteristics were consistent with those in the dog model (Figure 3).

B GAIT DEPENDENCE IN THE HORSE MODEL

Figures S2A–C show the gait dependence of the periodic solution that results from independently changing μ_0, k_0, and κ by $\pm 50\%$ from the horse parameter set ($\mu_0 = 0.72$, $k_0 = 2.2$, and $\kappa = 0.21$), where Sequences 1, 5, and 9 appeared for $\varepsilon_\mu = a\varepsilon_k$, $\varepsilon_\mu < a\varepsilon_k$, and $\varepsilon_\mu > a\varepsilon_k$, respectively. Although the coefficient a changed slightly when μ_0 and k_0 increased (Figures S2A, B, D, and E), it largely decreased as κ increased (Figures S2C and F). These characteristics were consistent with those in the dog model (Figure 4).

C DERIVATION OF SEQUENCE 1 CONDITION USING PERTURBATION THEORY

We derived the condition of ε_μ and ε_k ($\varepsilon_\mu = a\varepsilon_k$) to achieve Sequence 1 approximately using perturbation theory based on the approximate solution of the symmetric model ($\varepsilon_\mu = \varepsilon_k = 0$) analytically derived in (Adachi et al. (2020)). We used $\varepsilon_\mu = a\varepsilon$ and $\varepsilon_k = \varepsilon$ $(\varepsilon, a \geq 0)$ for $\varepsilon \ll 1$ and assumed the following periodic solution for Sequence 1:

$$z(\tau) = z_0(\tau) + \varepsilon z_1(\tau) + O(\varepsilon^2) \tag{S1a}$$

$$\theta(\tau) = \theta_0(\tau) + \varepsilon\theta_1(\tau) + O(\varepsilon^2) \tag{S1b}$$

$$\phi(\tau) = \phi_0(\tau) + \varepsilon\phi_1(\tau) + O(\varepsilon^2), \quad 0 \leq \tau \leq T, \tag{S1c}$$

where $z_0(\tau)$, $\theta_0(\tau)$, and $\phi_0(\tau)$ correspond to the periodic solution of the symmetric model (i.e., $\varepsilon = 0$) and T is the time duration from one apex to the next apex. We assumed $T = \tau_0^{\mathrm{f1}} + \tau_0^{\mathrm{ds}} + \tau_0^{\mathrm{f2}} + O(\varepsilon^2)$ based on the simulation results, where τ_0^{f1}, τ_0^{ds}, and τ_0^{f2} correspond to the time durations of the first flight, double stance, and next flight phases, respectively. We also assumed that the periodic solution satisfied the same condition $z(\tau_0^{\mathrm{f1}}) = z(\tau_0^{\mathrm{f1}} + \tau_0^{\mathrm{ds}})$ as that assumed in the symmetric model in our previous work (Adachi et al. (2020)).

By linearizing the governing equations under the assumptions $|\theta| \ll 1$ and $|\phi| \ll 1$, we reduced the equations of motion (3) to

$$\ddot{z}_0 + \sum_{i\in\mathcal{I},\, j\in\mathcal{J}} f_{ij}^0 + 1 + \varepsilon\left[\ddot{z}_1 + \sum_{i\in\mathcal{I},\, j\in\mathcal{J}} f_{ij}^1 + (1/a)k_0 \sum_{j\in\mathcal{J}} (f_{\mathrm{F}j}^0 - f_{\mathrm{H}j}^0)\right] + O(\varepsilon^2) = 0 \tag{S2a}$$

$$2\mu_0\ddot{\theta}_0 + \sum_{j\in\mathcal{J}} d_j(f_{\mathrm{F}j}^0 + f_{\mathrm{H}j}^0) + \varepsilon\Bigg[2\mu_0\ddot{\theta}_1 + \sum_{j\in\mathcal{J}} d_j(f_{\mathrm{F}j}^1 + f_{\mathrm{H}j}^1) + 2\mu_0\ddot{\phi}_0 + (1/a)k_0 \sum_{j\in\mathcal{J}} d_j(f_{\mathrm{F}j}^0 - f_{\mathrm{H}j}^0)\Bigg] + O(\varepsilon^2) = 0 \tag{S2b}$$

$$2\mu_0\ddot{\phi}_0 + \sum_{j\in\mathcal{J}} d_j(f_{\mathrm{F}j}^0 - f_{\mathrm{H}j}^0) + 4\kappa k_0\phi_0 + \varepsilon\Bigg[2\mu_0\ddot{\phi}_1 + \sum_{j\in\mathcal{J}} d_j(f_{\mathrm{F}j}^1 - f_{\mathrm{H}j}^1) + 4\kappa k_0\phi_1 + 2\mu_0\ddot{\theta}_0 + (1/a)k_0 \sum_{j\in\mathcal{J}} d_j(f_{\mathrm{F}j}^0 + f_{\mathrm{H}j}^0)\Bigg] + O(\varepsilon^2) = 0 \tag{S2c}$$

and reduced the conditions (5) and (6) for the phase transition and periodicity to

$$\dot{z}_0(0) + \varepsilon\dot{z}_1(0) + O(\varepsilon^2) = 0 \tag{S3a}$$

$$z_0(\tau_0^{\mathrm{f1}}) + \phi_0(\tau_0^{\mathrm{f1}}) + \varepsilon\left[z_1(\tau_0^{\mathrm{f1}}) + \phi_1(\tau_0^{\mathrm{f1}})\right] + O(\varepsilon^2) = 0 \tag{S3b}$$

$$\theta_0(\tau_0^{\mathrm{f1}}) + \varepsilon\left[\theta_1(\tau_0^{\mathrm{f1}})\right] + O(\varepsilon^2) = 0 \tag{S3c}$$

$$z_0(\tau_0^{\mathrm{f1}} + \tau_0^{\mathrm{ds}}) + \phi_0(\tau_0^{\mathrm{f1}} + \tau_0^{\mathrm{ds}}) + \varepsilon\left[z_1(\tau_0^{\mathrm{f1}} + \tau_0^{\mathrm{ds}}) + \phi_1(\tau_0^{\mathrm{f1}} + \tau_0^{\mathrm{ds}})\right] + O(\varepsilon^2) = 0 \tag{S3d}$$

$$\theta_0(\tau_0^{\mathrm{f1}} + \tau_0^{\mathrm{ds}}) + \varepsilon\left[\theta_1(\tau_0^{\mathrm{f1}} + \tau_0^{\mathrm{ds}})\right] + O(\varepsilon^2) = 0 \tag{S3e}$$

$$\dot{z}_0(\tau_0^{\mathrm{f1}} + \tau_0^{\mathrm{ds}} + \tau_0^{\mathrm{f2}}) + \varepsilon\left[\dot{z}_1(\tau_0^{\mathrm{f1}} + \tau_0^{\mathrm{ds}} + \tau_0^{\mathrm{f2}})\right] + O(\varepsilon^2) = 0 \tag{S3f}$$

$$q_0(0) - B_{\mathrm{LR}}\, q_0(\tau_0^{\mathrm{f1}} + \tau_0^{\mathrm{ds}} + \tau_0^{\mathrm{f2}}) + \varepsilon\left[q_1(0) - B_{\mathrm{LR}}\, q_1(\tau_0^{\mathrm{f1}} + \tau_0^{\mathrm{ds}} + \tau_0^{\mathrm{f2}})\right] + O(\varepsilon^2) = 0, \tag{S3g}$$

where

$$f_{ij}^k = \begin{cases} k_0\,(z_k + d_j(\theta_k + h_j\phi_k)) & \text{stance phase} \\ 0 & \text{swing phase} \end{cases} \qquad i = \mathrm{F}, \mathrm{H},\ j = \mathrm{L}, \mathrm{R},\ k = 0, 1$$

$q_i = [z_i\, \theta_i\, \phi_i\, \dot{z}_i\, \dot{\theta}_i\, \dot{\phi}_i]^{\mathrm{T}} (i = 0, 1)$, $h_{\mathrm{F}} = 1$, and $h_{\mathrm{H}} = -1$.

We obtained $z_0(\tau)$, $\theta_0(\tau)$, and $\phi_0(\tau)$ as follows:

$$z_0(\tau) = \begin{cases} z_0^{\mathrm{f1}}(\tau;p), & 0 \le \tau < \tau_0^{\mathrm{f1}} \\ z_0^{\mathrm{ds}}(\tau;p), & \tau_0^{\mathrm{f1}} \le \tau < \tau_0^{\mathrm{f1}} + \tau_0^{\mathrm{ds}} \\ z_0^{\mathrm{f2}}(\tau;p), & \tau_0^{\mathrm{f1}} + \tau_0^{\mathrm{ds}} \le \tau < \tau_0^{\mathrm{f1}} + \tau_0^{\mathrm{ds}} + \tau_0^{\mathrm{f2}} \end{cases} \tag{S4}$$

$$\theta_0(\tau) = 0 \quad 0 \le \tau < \tau_0^{\mathrm{f1}} + \tau_0^{\mathrm{ds}} + \tau_0^{\mathrm{f2}} \tag{S5}$$

$$\phi_0(\tau) = \begin{cases} \phi_0^{\mathrm{f1}}(\tau;p), & 0 \le \tau < \tau_0^{\mathrm{f1}} \\ \phi_0^{\mathrm{ds}}(\tau;p), & \tau_0^{\mathrm{f1}} \le \tau < \tau_0^{\mathrm{f1}} + \tau_0^{\mathrm{ds}} \\ \phi_0^{\mathrm{f2}}(\tau;p), & \tau_0^{\mathrm{f1}} + \tau_0^{\mathrm{ds}} \le \tau < \tau_0^{\mathrm{f1}} + \tau_0^{\mathrm{ds}} + \tau_0^{\mathrm{f2}}, \end{cases} \tag{S6}$$

where

$$\begin{aligned} p &= [z_0(0), \phi_0(0), \dot{\phi}_0(0)/\omega_{\mathrm{f}}, \nu_1, \nu_2, \omega_{\mathrm{f}}]^{\mathrm{T}} \\ \nu_{1,2} &= \tfrac{k_0}{2\mu_0}\left[1 + 2\mu_0 + 2\kappa \mp \sqrt{(1 + 2\mu_0 + 2\kappa)^2 - 16\mu_0\kappa}\right] \\ \omega_{\mathrm{f}} &= \sqrt{2\kappa k_0/\mu_0} \end{aligned}$$

$z_0^{\mathrm{f1}}(\tau;p)$, $z_0^{\mathrm{ds}}(\tau;p)$, $z_0^{\mathrm{f2}}(\tau;p)$, $\phi_0^{\mathrm{f1}}(\tau;p)$, $\phi_0^{\mathrm{ds}}(\tau;p)$, and $\phi_0^{\mathrm{f2}}(\tau;p)$ correspond to the periodic solution of the symmetric model ($\varepsilon = 0$) obtained in (Adachi et al. (2020)).

We obtained $z_1(\tau)$, $\theta_1(\tau)$, and $\phi_1(\tau)$ as follows:

$$z_1(\tau) = 0 \quad 0 \le \tau < \tau_0^{\mathrm{f1}} + \tau_0^{\mathrm{ds}} + \tau_0^{\mathrm{f2}} \tag{S7}$$

$$\theta_1(\tau) = \begin{cases} -A_{\mathrm{f}} \sin(\omega_{\mathrm{f}}\tau) + \dfrac{A_{\mathrm{f}} \sin(\omega_{\mathrm{f}}\tau_0^{\mathrm{f1}})}{\tau_0^{\mathrm{f1}}}\tau, & 0 \le \tau < \tau_0^{\mathrm{f1}} \\ A_\theta \sin(\omega_\theta(\tau - \tau_0^{\mathrm{f1}})) + \displaystyle\sum_{i=1,2} \frac{N_i}{\omega_i^2 - \omega_\theta^2} \sin(\omega_i(\tau - \tau_0^{\mathrm{f1}}) + \psi_i) - \frac{C}{\omega_\theta^2}, & \tau_0^{\mathrm{f1}} \le \tau < \tau_0^{\mathrm{f1}} + \tau_0^{\mathrm{ds}} \\ -A_{\mathrm{f}} \sin(\omega_{\mathrm{f}}(\tau - (\tau_0^{\mathrm{f1}} + \tau_0^{\mathrm{ds}} + \tau_0^{\mathrm{f2}}))) + \dfrac{A_{\mathrm{f}} \sin(\omega_{\mathrm{f}}\tau_0^{\mathrm{f2}})}{\tau_0^{\mathrm{f2}}}(\tau - (\tau_0^{\mathrm{f1}} + \tau_0^{\mathrm{ds}} + \tau_0^{\mathrm{f2}})), & \tau_0^{\mathrm{f1}} + \tau_0^{\mathrm{ds}} \le \tau < \tau_0^{\mathrm{f1}} + \tau_0^{\mathrm{ds}} + \tau_0^{\mathrm{f2}} \end{cases} \tag{S8}$$

$$\phi_1(\tau) = 0 \quad 0 \le \tau < \tau_0^{\mathrm{f1}} + \tau_0^{\mathrm{ds}} + \tau_0^{\mathrm{f2}}, \tag{S9}$$

where

$$
\begin{aligned}
A_{\mathrm{f}} &= \dot{\phi}_0(0)/\omega_{\mathrm{f}} \\
\omega_\theta &= \sqrt{k_0/\mu_0} \\
N_i &= G_i(((1/a)-1-2\kappa)\nu_i + 4\kappa k_0)/(4\mu_0) \quad i=1,2 \\
G_i &= \sqrt{A_i^2+B_i^2} \quad i=1,2 \\
A_i &= \zeta_i+\rho_i \quad i=1,2 \\
B_i &= \eta_i/\omega_i \quad i=1,2 \\
\rho_{1,2} &= \pm 2(\nu_{2,1}-2k_0)/(\nu_{1,2}(\nu_2-\nu_1)) \\
\zeta_{1,2} &= \tfrac{2}{\nu_2-\nu_1}\left[\pm(\nu_{2,1}-2k_0)z_0(\tau_0^{\mathrm{f1}}) \mp 2k_0\phi_0(\tau_0^{\mathrm{f1}})\right] \\
\eta_{1,2} &= \tfrac{2}{\omega_i(\nu_2-\nu_1)}\left[\pm(\nu_{2,1}-2k_0)\dot{z}_0(\tau_0^{\mathrm{f1}}) \mp 2k_0\dot{\phi}_0(\tau_0^{\mathrm{f1}})\right] \\
\omega_i &= \sqrt{\nu_i} \quad i=1,2 \\
\sin\psi_i &= A_i/\sqrt{A_i^2+B_i^2} \quad i=1,2 \\
\cos\psi_i &= B_i/\sqrt{A_i^2+B_i^2} \quad i=1,2 \\
C &= (1/4\mu_0)\left[2\kappa\left((\nu_1-2k_0)\rho_1+(\nu_2-2k_0)\rho_2\right) - ((1/a)-1)(\nu_1\rho_1+\nu_2\rho_2)\right].
\end{aligned}
$$

We obtained A_θ and a by solving the following equations:

$$\sum_{i=1,2}\frac{N_i}{\omega_i^2-\omega_\theta^2}\sin\psi_i = \frac{C}{\omega_\theta^2} - A_\theta\sin\psi_\theta \tag{S10a}$$

$$\sum_{i=1,2}\frac{\omega_i N_i}{\omega_i^2-\omega_\theta^2}\cos\psi_i = -\omega_{\mathrm{f}}A_{\mathrm{f}}\cos(\omega_{\mathrm{f}}\tau^{\mathrm{f}}) + \frac{A_{\mathrm{f}}\sin(\omega_{\mathrm{f1}}\tau_0^{\mathrm{f1}})}{\tau_0^{\mathrm{f1}}} - \omega_\theta A_\theta\cos\psi_\theta, \tag{S10b}$$

where $\psi_\theta = (\pi - \omega_\theta\tau_0^{\mathrm{ds}})/2$. As a result, we obtained $a \in \mathbb{R}^+$ uniquely and obtained the condition $\varepsilon_\mu = a\varepsilon_k$ for Sequence 1.

We compared the results of a obtained by the approximate analysis above and numerical simulation using the dog and horse models (Figure S3A). As shown in the figure, the approximate analysis almost reproduced the simulation results in both models. We also compared the dependence of a on the parameters μ_0, k_0, and κ between the approximate analysis and numerical simulation in the dog and horse models (Figures S3B and C, respectively). The approximate analysis reproduced similar tendencies to those of the numerical simulation for μ_0 and κ. However, they had some differences, mainly because of linearization errors.

REFERENCES

Adachi, M., Aoi, S., Kamimura, T., Tsuchiya, K., and Matsuno, F. (2020). Body torsional flexibility effects on stability during trotting and pacing based on a simple analytical model. *Bioinspir. Biomim.* 15, 055001

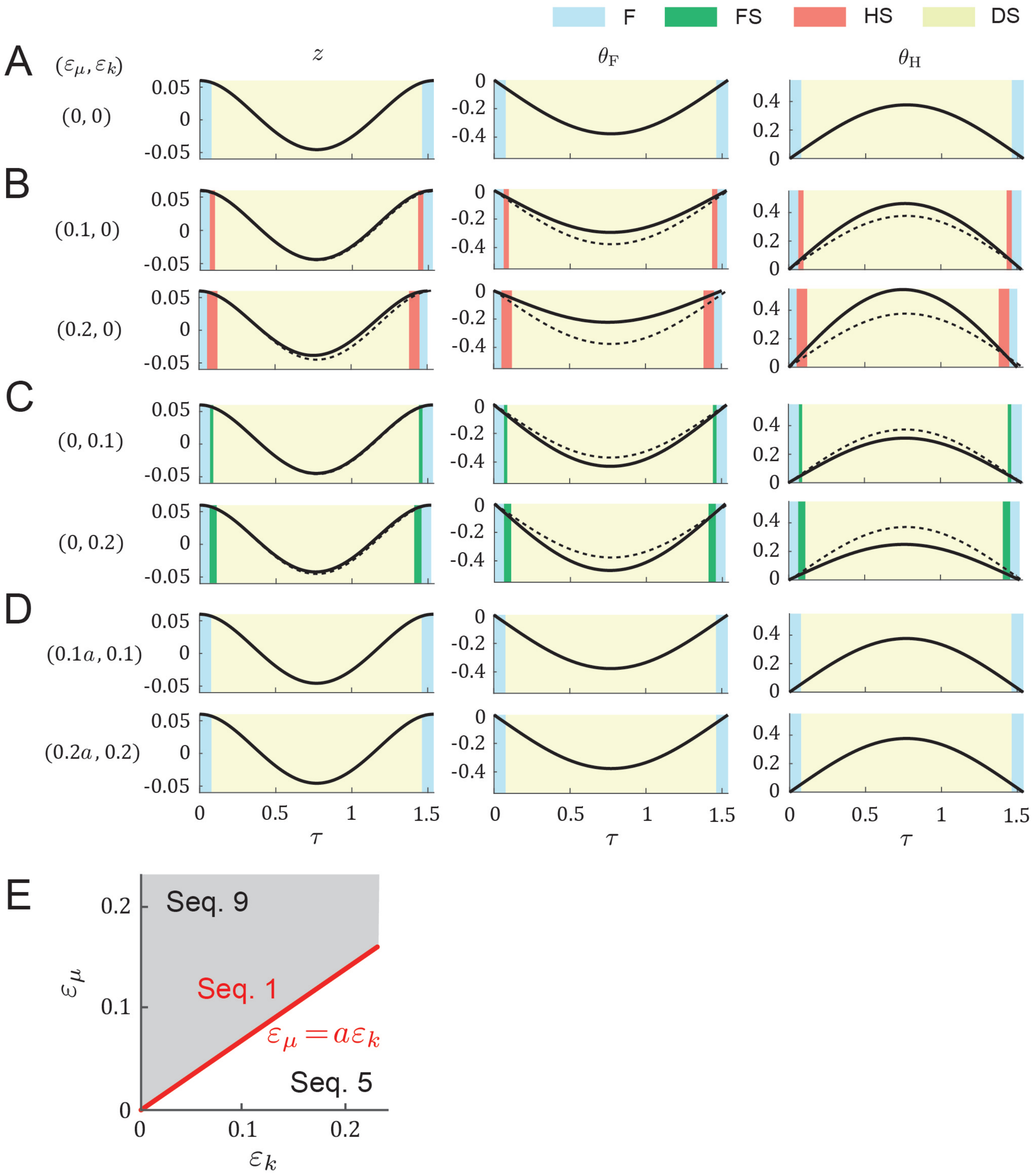

Figure S1. Gait dependence on ε_k and ε_μ in the horse model. Time profile of the periodic solution (A) for the symmetrical model ($\varepsilon_k = \varepsilon_\mu = 0$) and those for two values of (B) ε_μ with $\varepsilon_k = 0$, (C) ε_k with $\varepsilon_\mu = 0$, and (D) ε_k with $\varepsilon_\mu = a\varepsilon_k$. Cyan, green, pink, and yellow regions indicate flight (F), fore stance (FS), hind stance (HS), and double stance (DS), respectively. Dotted lines indicate the periodic solution of the symmetrical model. (E) Gait dependence on ε_k and ε_μ.

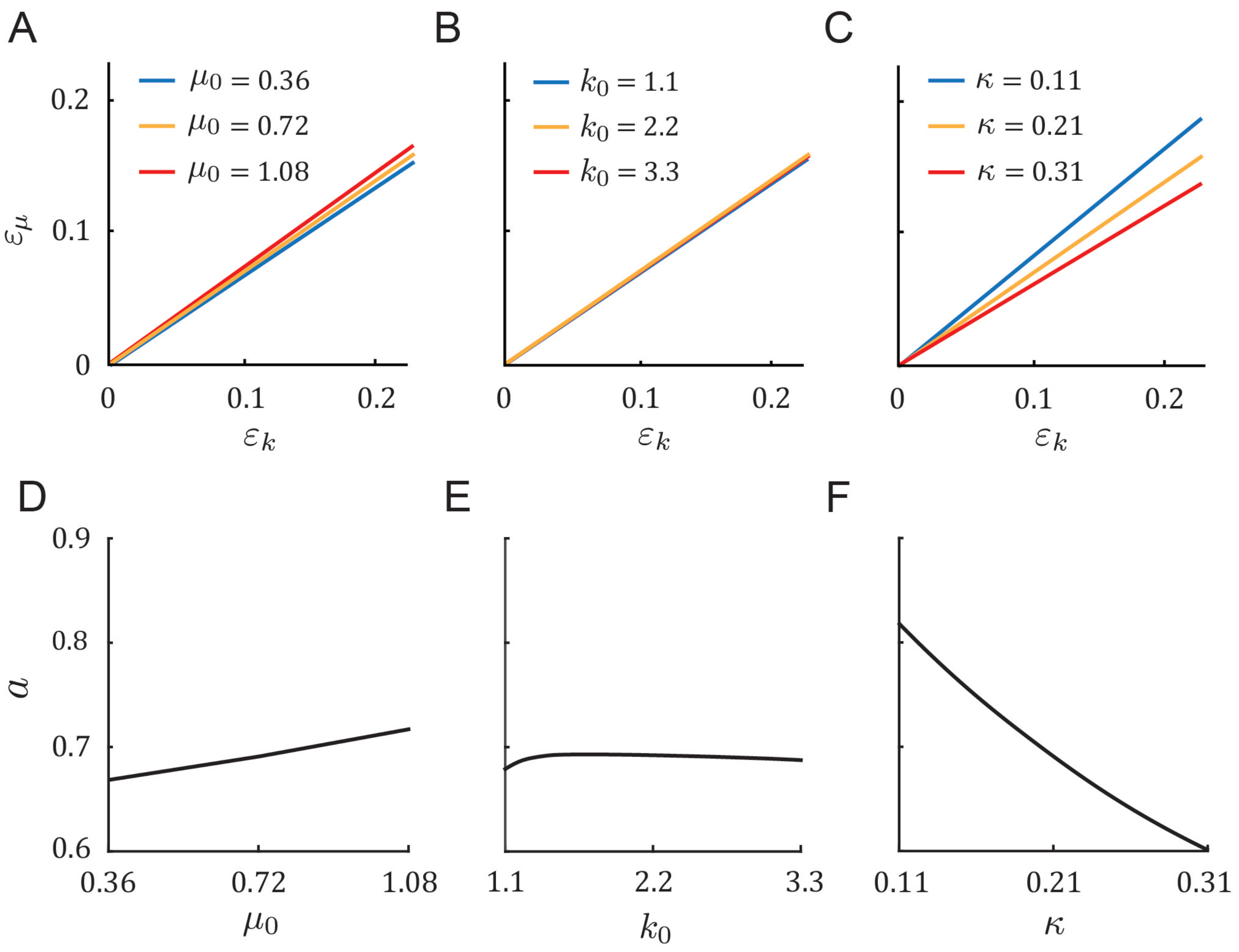

Figure S2. Gait dependence on physical parameters in the horse model. Condition of ε_k and ε_μ ($\varepsilon_\mu = a\varepsilon_k$) to achieve Sequence 1 for three values of (A) μ_0, (B) k_0, and (C) κ, while holding the other parameters constant at $\mu_0 = 0.72$, $k_0 = 2.2$, and $\kappa = 0.21$. Sequences 5 and 9 appeared when $\varepsilon_\mu < a\varepsilon_k$ and $\varepsilon_\mu > a\varepsilon_k$, respectively. Dependence of a on (D) μ_0, (E) k_0, and (F) κ.

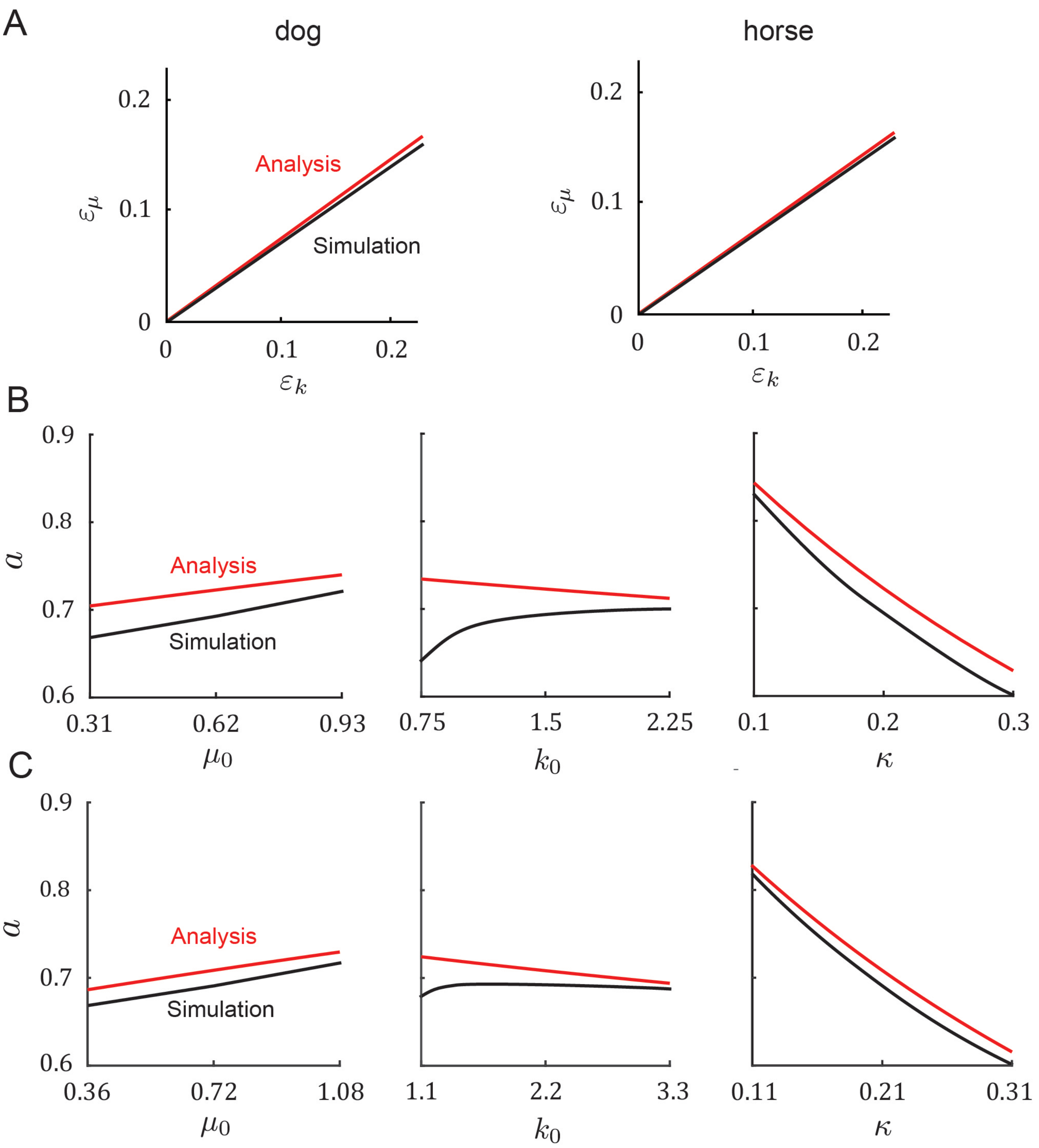

Figure S3. Comparison of a obtained using approximate analysis and numerical simulation. (A) Condition of ε_μ and ε_k ($\varepsilon_\mu = a\varepsilon_k$) to achieve Sequence 1 using dog and horse models. (B) Dependence of a on μ_0, k_0, and κ in the dog model. (C) Dependence of a on μ_0, k_0, and κ in the horse model.
